# Virucidal and Bactericidal Properties of Biocompatible Copper Textiles

**DOI:** 10.1002/gch2.202400346

**Published:** 2025-01-27

**Authors:** Andrei‐Florin Sandu, Sofya Danilova, Lauren Acton, Andrew Cobley, Phillip Gould

**Affiliations:** ^1^ Coventry University Group: Coventry University Whitefriars St Coventry 2706 UK

**Keywords:** bactericidal, biocompatible copper textile, electroless copper plating on textile, global health and healthcare, virucidal

## Abstract

The COVID‐19 pandemic highlights the global threat posed by emerging viruses, emphasizing the critical need for effective strategies to combat pathogen transmission. Moreover, alongside emerging viruses, the increasing threat of antimicrobial resistance further reinforces the need to develop novel methods for infection control. Anti‐pathogenic coatings on textiles offer a promising solution; in this study, three electroless copper‐plated fabrics are evaluated for their antipathogenic properties following International Standards Organisation (ISO) standards. Prior to electroless plating, materials are activated either by immersion in a Pd catalyst solution (material A) or by ink‐jet printing Cu/Ag catalyst along the weft (material B) or warp thread (material C). This study demonstrates that activation method influences the materials antipathogenic performance, with all materials achieving complete bactericidal/fungicidal neutralization within 30 min of incubation. Material B exhibits up to 4‐log virucidal effects within 1 h against viruses such as coronavirus (OC43, 229E), Influenza A (H1N1), and Rotavirus A. Furthermore, biocompatibility testing indicates that material B exhibited low in vitro cytotoxicity. Textile B demonstrates strong antibacterial results even after one year of accelerated aging with no significant difference (*P* = 0.74) in efficiency against MRSA, highlighting promising applications for infection control in clinical settings reducing pathogen transmission, nosocomial infections and the associated economic burden.

## Graphical abstract

The study demonstrates that metalized materials neutralize pathogens effectively upon contact. Within 1 h, they achieve antipathogenic effects, neutralizing bacteria completely and viruses up to 4‐log. Even after one year of aging, the material maintains its antibacterial properties. Additionally, they exhibit low toxicity with human skin cells, making them a promising solution for infection control in different environments.

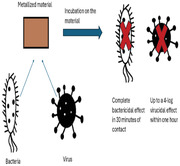

## 1 Introduction

Respiratory viruses are a significant contributor to global mortality and morbidity, this was further heightened by the COVID‐19 pandemic, which, as of January 2025, resulted in over 777 million reported cases and 7 million deaths.^[^ ^1^ ^]^ While viral infections are a major global health concern, with more than 178 million cases worldwide, bacterial infections have an even greater impact, accounting for more than 415 million infections (note: cumulative data for total viral or bacterial infections are not available in the literature).^[^ ^2^ ^]^ Additionally, antimicrobial resistance (AMR) is a critical issue, responsible for approximately 4.95 million deaths each year; The World Bank projects that by 2050 AMR could lead to an additional healthcare cost of one trillion dollars.^[^ ^3^ ^]^ *Staphylococcus aureus* is a significant cause of healthcare‐associated infections (HAI). In the United States, the incidence of *S. aureus* infections is estimated at 20.1 cases per 100 000 population, with a mortality rate of 3.2 (CDC),^[^ ^4^ ^]^ and *Pseudomonas aeruginosa* individually account for more than 10% of nosocomial infections.^[^ ^5^ ^]^ Untreated infections leading to sepsis affect 1.7 million adults in the U.S. annually, (CDC).^[^ ^6^ ^]^ According to the CDC, diarrheagenic *Escherichia coli* is responsible for an estimated 111 million illnesses and 63000 deaths globally each year.^[^ ^7^ ^]^ In the United States, foodborne pathogens such as *E. coli* and *Salmonella* contribute to approximately 9.4 million infections annually.^[^ ^8^ ^]^ These pathogens continue to pose significant health risks, resulting in frequent and severe outbreaks.^[^ ^9^ ^]^ However, these risks could be mitigated with the application of surface coatings designed to neutralize pathogens.^[^ ^10^ ^]^

Global population movement and the rapid evolution of transportation pathways facilitate the swift transmission of pathogens worldwide.^[^ ^11^ ^]^ This is evident with both SARS‐CoV‐2 and HIV where it is reported that increased human migration between the mid‐1970s and 1980 led to an estimated 100 000 to 300 000 HIV infections.^[^ ^12^ ^]^ Higher population and urbanization demography directly influence the trading sector which facilitates pathogen spread and hotspot generation followed by translocation beyond the ecological and geographical boundaries.^[^ ^12^ ^]^ For instance, Cholera and Variant Creutzfeldt–Jakob disease (vCJD) transmission.^[^ ^13^ ^]^ Environmental changes, including climate change drive pathogenic mutations,^[^ ^14^ ^]^ altering species dynamics, expanding pathogen range into new areas, as emphasized for tuberculosis, West Nile virus and many respiratory viruses.^[^ ^15^, ^16^, ^17^ ^]^ Human habitat expansion, agriculture, and livestock intensification further promote pathogen transmission.^[^ ^16^ ^]^ This is further exacerbated by intense antibiotic use and poor livestock conditions, which all contribute to bacterial resistance selection, as observed in multiple bacterial species such as *Salmonella, Staphylococcus, and E. coli*.^[^ ^18^ ^]^

Pathogen transmission in hospitals and other care facilities primarily occurs through airborne routes and direct or indirect contact,^[^ ^19^ ^]^ despite the usage of conventional surface cleaning methods, which often yield suboptimal results.^[^ ^20^ ^]^ Hospital textiles, such as linens and white coats, can harbor bodily fluids, promoting microorganism growth and the spread of infections, with a challenging process flow required for effective decontamination.^[^ ^21^ ^]^ Pathogens like MRSA (Methicillin‐Resistant *Staphylococcus aureus*), influenza, and norovirus pose significant challenges, with about 1 in 31 hospitalized patients acquiring infections, resulting in $28.4 billion in healthcare costs annually.^[^ ^22^, ^23^, ^24^, ^25^ ^]^ Patient movement within and between hospital units further contributes to the spread of pathogens as seen for Multidrug‐resistant *Enterobacteriaceae*.^[^ ^26^ ^]^ For example, during hospital admissions or triage multiple photogenic bacteria with AMR genes were identified such as *E. coli*, *K. pneumoniae*, *Enterobacter spp*. and *Citrobacter spp*.^[^ ^27^ ^]^ Outpatient clinics also play a role, with about 46% of Human parainfluenza virus (HPIV) cases occurring during nosocomial outbreaks linked to these facilities.^[^ ^28^ ^]^ Respiratory syncytial virus (RSV) and rhinovirus are significant contributors to neonatal and pediatric admissions and contribute to HAI due to their high transmissibility.^[^ ^29^, ^30^ ^]^ Preventing pathogen spread is crucial, and one effective strategy is using virucidal/bactericidal coatings, which significantly reduce contamination and infection risks.^[^ ^31^ ^]^ For instance, hospital bed rails metalized with antimicrobial materials showed an 88.9% reduction in *Staphylococcus* spp. populations compared to untreated controls.^[^ ^32^ ^]^

Copper is well known for its bactericidal and virucidal properties, via the formation of reactive oxygen species (ROS), disruption of respiratory or enzymatic metabolism and interference or damage of cell membranes, proteins, and genetic material.^[^ ^33^ ^]^ Multiple studies show that copper‐impregnated textiles reduce pathogenic transmission in hospitals.^[^ ^32^, ^34^, ^35^, ^36^ ^]^ In this study, electroless copper‐plated textiles have been tested against a wide range of bacteria/fungi (12 species) and viruses (6 species). To our knowledge, the previous works usually focus on testing against a narrower range (up to 4 species) of bacteria or viruses.^[^ ^37^, ^38^ ^]^ Prior to electroless copper plating, the textile must be activated by depositing a catalyst on its surface. It has been previously reported that the direction of catalyst deposition on textile prior to electroless plating can affect the mechanical and electrical properties of the copper layer.^[^ ^39^ ^]^ In this study, we demonstrate that the antibacterial and antiviral properties are also influenced by how the material is activated prior to electroless plating. A better understanding of the dependence between the material fabrication method and its final properties will help identify the most effective manufacturing method to produce textile materials with the strongest antibacterial and virucidal properties.

### 1.1 Aims

This study investigates the comparative properties of textiles metalized through electroless plating, with a focus on their potential as antimicrobial surfaces. Prior to metalization, textile samples were activated by either immersion in a Pd based catalyst solution or by ink‐jet printing Cu/Ag catalyst along the weft or warp threads. The impact of textile orientation during catalyst deposition on virucidal and bactericidal efficacy was assessed, followed by biocompatibility according to International Standards Organisation (ISO) standards. This research aims to advance the development of versatile, biocompatible materials capable of reducing pathogen transmission, with broad applications in both clinical and non‐clinical environments.

## 2 Experimental Section

### 2.1 Textiles Samples Preparation

**Figure 1:**
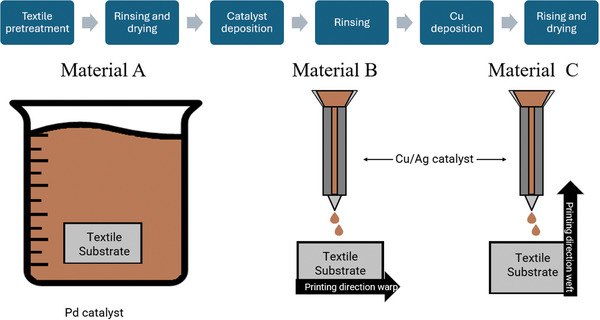
Schematic description of stages of electroless copper plating process on textile and textile sample preparation. Material A is based on bulk immersion of the textile into the Pd catalyst solution. For materials B and C, the catalyst ink was printed on the textile substrate.

The polyester textile was purchased from Whaleys Ltd. (Bradford, UK). The thickness of the textile was 0.32 mm, mass per unit area—120 g m^−2^, ends per centimeter—69, and picks per centimeter is 30. The textile was covered with a copper metal layer using the electroless plating process.^[^ ^39^, ^40^ ^]^ This process consists of three stages (**Figure** **1**): pre‐treatment, catalyst deposition, and metal deposition.

During pre‐treatment, the textile samples were immersed in commercially available Circuposit Conditioner 3323A for 5 min at 50 °C. All Circuposit solutions were prepared according to the manufacturer's instructions provided in the data sheets. After conditioning, the samples were rinsed in reverse osmosis‐cleaned water for 5 min and dried in an oven at 45 °C for at least 12 h. Catalyst deposition on the samples was carried out using the following methods. Material A (Figure 1) was immersed in commercially available Circuposit Pre‐dip 3340 at 25 °C for 1 min, followed by immersion in Circuposit Catalyst 3344 solution for 5 minutes at 40 °C. The Pre‐dip is an acidic solution used to protect subsequent catalyst bath from water contamination. The catalyst solutions contain a Sn/Pd catalyst and were prepared at a concentration of 2%, according to the technical data sheet provided by A‐Gas Electronic Materials. The undiluted catalyst component contains 8 g L^−1^ of palladium chloride. For simplicity, this catalyst solution will be referred to as the Pd catalyst.

For materials B and C (Figure 1) the catalyst was deposited via ink‐jet printing using 5% Cu/Ag based nanoparticle ink. Details of the ink preparation and deposition conditions can be found in the article by Azar et al.^[^ ^39^ ^]^ For simplicity, this catalyst solution will be referred to as the Cu/Ag catalyst. The Cu/Ag catalyst was ink‐jet printed three times on one side of the sample to form a rectangle with 15 × 7 cm dimensions. The printing process was conducted line by line from left to right. A textile consists of parallel warp yarns, around which the weft yarns are threaded. Material B was prepared with the Cu/Ag catalyst deposited parallel to the warp threads, whereas Material C was prepared by depositing the Cu/Ag catalyst parallel to the weft threads.^[^ ^39^ ^]^

After catalyst deposition, material A was immersed in reverse osmosis‐cleaned water for 5 min at 20 °C. Then, all materials were placed in commercially available Circuposit 3350‐1 electroless Cu solution, which contains Cu salt, NaOH, and formaldehyde. The samples were immersed for 25 min at 46 °C.

After the metal deposition process, all samples were washed with reverse osmosis‐cleaned water for five minutes and dried in an oven at 50 °C overnight. Dried samples were then cut into 2 × 2 cm pieces.^[^ ^39^, ^40^ ^]^ All metalized materials and unmetalized control materials (negative control) were UV sterilized in a biosafety hood (BLS‐2) for one hour prior to testing.

### 2.2 Material Characterization

A Sigma 500 VP scanning electron microscope was used to obtain images of the metalized textiles. An X‐MaxN 80 Oxford Instruments silicon drift detector, fitted in the SEM, was used to map the copper distribution across the selected sample area.

### 2.3 Material Aging

Material aging was accomplished via accelerated heating following the equation.^[^ ^41^ ^]^ The control material and metalized materials were aged to the equivalent of one year at 65 °C for a 20 d incubation period. Following material aging, materials were tested in biological triplicates against MRSA using the agar diffusion method. A paired t‐test was used via Prism software (version 9.5.0) to determine the statistical difference in antibacterial activity between the aged material and unaged material.

### 2.4 Microbiology

#### 2.4.1 Microbial Culture

Microbiological isolates were purchased from ATCC and NCTC (Table S1, Supporting Information), and all 12 isolates were grown according to the manufacturer's recommendations and in compliance with ISO standard BS EN ISO 20743:2021.

Overnight bacterial or fungal cultures used for the experiments were adjusted accordingly against McFarland Standards of 0.5 Modified Fishman Units (MFU), with a cell count density of 1.5 × 10^8^.^[^ ^42^ ^]^ Each experiment was performed in biological triplicates with technical duplicates.

#### 2.4.2 Agar Diffusion Susceptibility Test

The agar diffusion susceptibility test protocol was followed with the corresponding alterations.^[^ ^43^ ^]^ Overnight cultures adjusted to 0.5 MFU were spread over the surface of an agar plate using a sterile swab. Metalized polyester and control materials were directly placed on the surface of the bacterial lawn (no closer than 24 mm) using sterile forceps and results were recorded as a zone of inhibition (ZOI) (mm) after 18 h of incubation.^[^ ^43^ ^]^

#### 2.4.3 Antimicrobial Direct Material Contact Testing

Direct material contact testing was performed as BS EN ISO 20743:2021, with the following alterations. Each material was inoculated with 0.5 MFU of each bacterial culture and incubated for 30 minutes at room temperature. After the incubation period, the bacterial specimens were recovered directly from the material surface by washing the material with 500 µL of SCDLP media. The minimum bactericidal/fungicidal concentration technique (MBC/MFC) was used for the recovered samples to establish the bactericidal activity of the materials tested.^[^ ^44^ ^]^ The bactericidal/fungicidal results were assessed based on the growth of the harvested specimens after 18 h of incubation on specimen‐specific growth media.^[^ ^43^ ^]^

### 2.5 Cell Culture

All cell lines were purchased from ATCC and were cultured according to the manufacturer's recommendations (Table S2, Supporting Information).

### 2.6 Virology

#### 2.6.1 Viral Propagation

Viral propagation was performed according to specimen growth requirements (Table S3, Supporting Information). Viral harvesting was performed when CPE (Cytopathic effect) in cell lines was higher than 60%. Each flask was harvested by using sterile glass beads (2 mm), followed by centrifugation at 100x*g* for 5 min. All viral stocks were kept at −80 °C and were only used for one freeze‐thaw cycle.

#### 2.6.2 Viral Titration

Infectious viral titers were determined by plaque assays.^[^ ^45^ ^]^ Results were recorded after 5 d of incubation, plates were fixed with 4% (v/v) paraformaldehyde and stained with 1% (w/v) crystal violet, the viral concentration results being visually interpreted as plaque forming units (PFU).^[^ ^46^ ^]^

#### 2.6.3 Antiviral Testing

The viral samples used for testing were no higher than 10^6^ PFU/ml, according to BS EN ISO 18184:2019. Five hundred microliters of the viral sample were added directly to the metalized material and control material. Materials were incubated for one hour at room temperature. The recovered samples were titrated via the plaque assay.

Each viral experiment was performed according to BS EN ISO 18184:2019 in biological triplicates followed by four technical replicates. For OC43, HPIV‐3, and PR8 strains/variants, testing was repeated to ensure no false positive results were recorded and no viral discrepancy between tests was observed.

Viral stability was established for three viral specimens (OC43, PR8 and HPIV‐3) to ensure no environmental factors influenced potential reductions to the viral titer. Following the ISO 18184:2019 protocol the viral species were tittered prior to and after one hour of incubation at room temperature on the control polyester material. Following the ISO equation, the viral stability was accomplished as follows: the initial titer logarithm (L_pi_) was reduced from the logarithmic value of species after 1 h of incubation (L_ai_) on the control material. The logarithmic difference recorded should not be higher than 0.2, as higher values represent significant titer loss in the incubation process and are not reflective of any surface treatment.

### 2.7 Cytotoxicity

#### 2.7.1 Cytotoxicity Testing

All metalized materials and controls were tested in biological triplicate and technical duplicate in the HEKa (normal primary Human Epidermal Keratinocytes‐adult) cell line according to BS EN ISO standard 10993‐5:2009, following indirect contact cytotoxicity testing via agar diffusion method with incubation time modified for 2 h.

For the positive control, neat Distel was used, whereas the unmetalized control material was used as a negative control. The cell monolayer was fixed with 4% (v/v) paraformaldehyde and stained with 1% (w/v) crystal violet. Results were interpreted and assessed as a qualitative and quantitative test according to BS EN ISO:10993‐5‐2009, based on the reactivity of the cell monolayer.

The Cytation 5 cell imaging multimode reader with Gen5+ software (version 3.16.10) (Agilent Technologies, Stockport, UK) at × 20 magnification was used to capture, and cell count the monolayer after fixation and crystal violet staining. A one‐way ANOVA test was used via Prism software to establish the significant difference between populations.

#### 2.7.2 Confocal Microscopy in 2D Culture

For confocal microscopy, the indirect cytotoxicity method was followed. For confocal visualization, cell culture was performed in 35 mm Nunc Glass Bottom Dishes (cat: 150680) following the supplier guidelines.

HEKa cells were grown in 35 mm Glass Bottom Dishes for 18 h prior to testing. After the indirect method, cells were fixed with 4% (v/v) paraformaldehyde and washed with PBS. The cells were stained with CellBrite Cytoplasmic Membrane Dyes (549/565 nm) (cat: 30021), Live‐or‐Dye Fixable Viability Staining Kits (405/452 nm) (cat: 32018) and the primary antibodies, Human Cytokeratin 14, and Human Cytokeratin 19 Alexa Fluor 488‐conjugated Antibody (cat: MAB3164 and IC3506G). After 1 h of incubation, cells were washed twice with PBS and stained with secondary antibody APC (640/650 nm) (cat: 17‐4210‐82). The cells were incubated for a further hour and washed three times with PBS. Samples were visualized and recorded under the Nikon A1 LFOV camera under a Nikon Eclipse Ti2E microscope, using laser wide‐field fluorescence scan confocal Galvano, scan direction One way under Plan Apo Î» 100x Oil magnification and analyzed via NIS‐Elements confocal software. The average intensity of the 2D models was established via the Nikon software and a one‐way ANOVA test was used via Prism software to establish the significant difference between populations.

#### 2.7.3 Confocal Microscopy and 3D Culture

For 3D models, the SeedEZ 3D Cell Culture System (cat: Z742248‐24EA) model was used following the manufacture protocol with HEKa cells. Testing was accomplished by direct contact cytotoxicity testing method, directly incubating the materials for 2 h on the 3D scaffold.

The 3D culture was established by using SeedEZ 3D Cell Culture Scaffold. All scaffolds were fixed and stained as stated previously. Samples were visualized and recorded via the Ti2 microscope and Nikon A1 LFOV camera at Plan Apo λ 20 × magnification under laser wide‐field fluorescence, scan confocal Galvano, Bi‐Direction scan, the average maximum intensity of Z axis layers was combined by Nikon NIS‐Elements confocal software (version 6.10.01).

### 2.8 Statistical Analysis

Pre‐processing of data was performed for plaque assay results by following the plaque equation,^[^ ^47^ ^]^ Prism software (version 9.5.0), Gen5+ (version 3.16.10) software and Nikon NIS‐Elements confocal software (version 6.10.01). Data presentation was accomplished via Nikon NIS‐Elements confocal software and Prism software by performing mean and SD of the data. Statistical testing was accomplished via Prism software for all data sets. The following statistical tests were performed: for material aging data a Paired t‐test (P two‐tailed < 0.05 and *n* = 3), for cytotoxicity testing the overall cell count was analyzed and compared against the control material using ordinary one‐way ANOVA (*n* = 3 and *P* < 0.05) followed by Sidak post‐hoc test and the average intensity of the 2D images were analyzed via the ordinary one‐way ANOVA by comparing the groups against the control material (*n* = 3 and *P* < 0.05) followed by the post‐hoc Dunnett's test.

## 3 Results

### 3.1 Antibacterial Activity of Metalized Materials

#### 3.1.1 Agar Diffusion Susceptibility Testing

The agar diffusion susceptibility test was performed against a panel of 12 clinically relevant microbial isolates (Table S1, Supporting Information) to establish the antibacterial/antifungal activity of the metalized materials.

**Figure 2:**
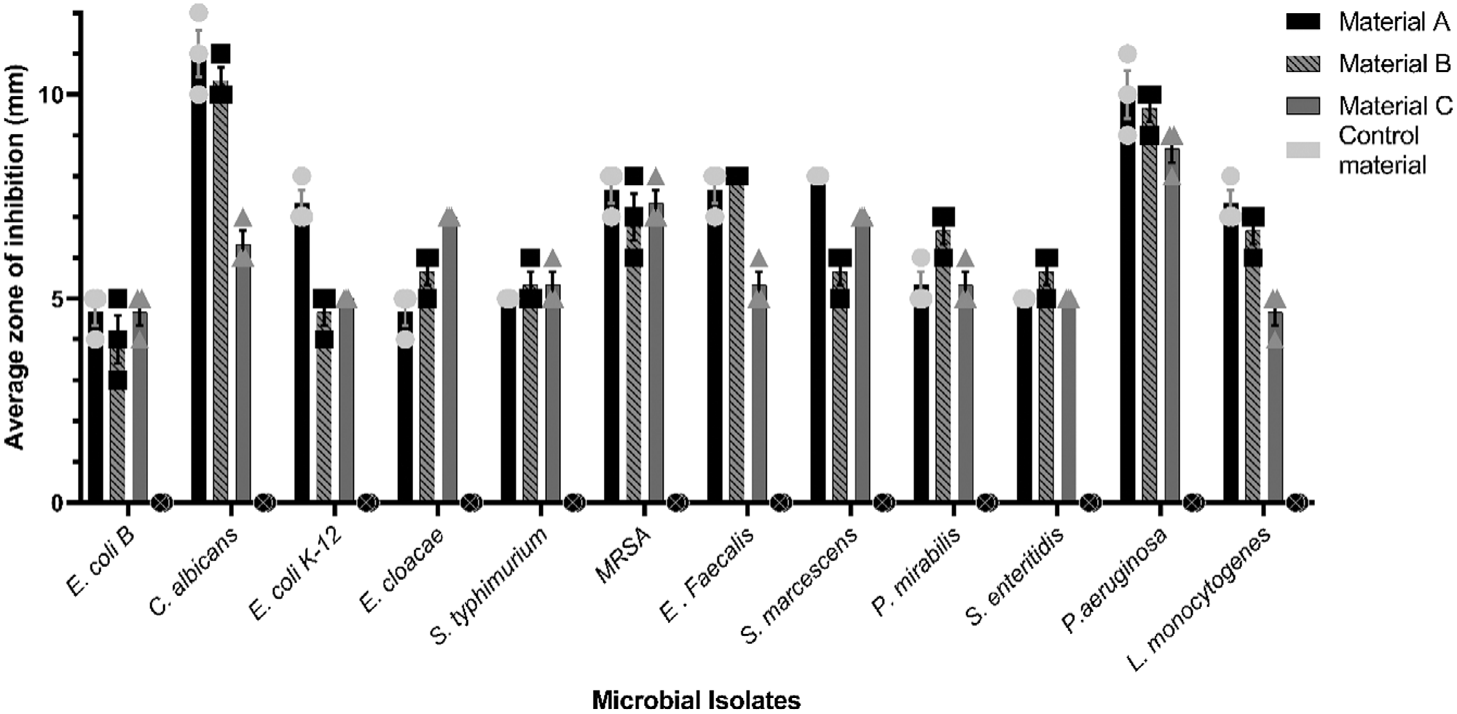
Agar diffusion susceptibility testing results for 12 microbial isolates against the three metalized materials and the polyester control material. The data is presented as the mean measurement of inhibition for each isolate with standard deviation (mean ± SD). The control material displayed no inhibition zones across all microbial isolates and biological replicates. The dots, squares and triangles represent the values of each biological replicate (*n* = 3).

After 18 hours of incubation, results showed that all bacterial/fungal specimens presented a zone of inhibition around the metalized materials, whereas the control material showed no zone of inhibition (**Figure** **2**). The lowest average zone of inhibition value was marked by material B against *E. coli* B and the largest inhibition value recorded was for material A and B with *P. aeruginosa* and *C. albicans*. Material A had the highest overall average inhibition of 6.97 mm (SD = 2.10), followed by material B with 6.61 mm (SD = 1.93) and with the lowest activity material C with 5.97 mm (SD = 1.29) (Figure 2). By subtracting the standard deviation of each specimen's results from the average zone of inhibition for each material, it was found that materials B and C both yielded identical results, with an overall zone of inhibition of 4.77 mm.

#### 3.1.2 Antimicrobial Direct Material Contact Testing

The metalized materials were further tested by direct material contact testing with broth culture to establish the bacteriostatic or bactericidal effect. After 30 minutes of material contact with an average cell count density of 1.5 × 10^8^, the results show direct fungicidal/bactericidal activity for materials A, B and C, and no specimen could be recovered with the SCLDP media (data not shown). The control material presents microbial growth for all isolates tested.

### 3.2 Virucidal Activity of Metalized Materials

To ensure no reduction in the viral titer was due to the experimental model used, the viral titer of three representative viruses was calculated pre‐ and post‐1 h incubation with control material contact. All three viruses tested (OC43, HPIV‐3, and PR8) present values lower or equal to 0.2. The titers of all three viruses were unaffected by the 1 h incubation, demonstrating that any reduction in viral titer would be the consequence of exposure to the metalized materials.

**Figure 3:**
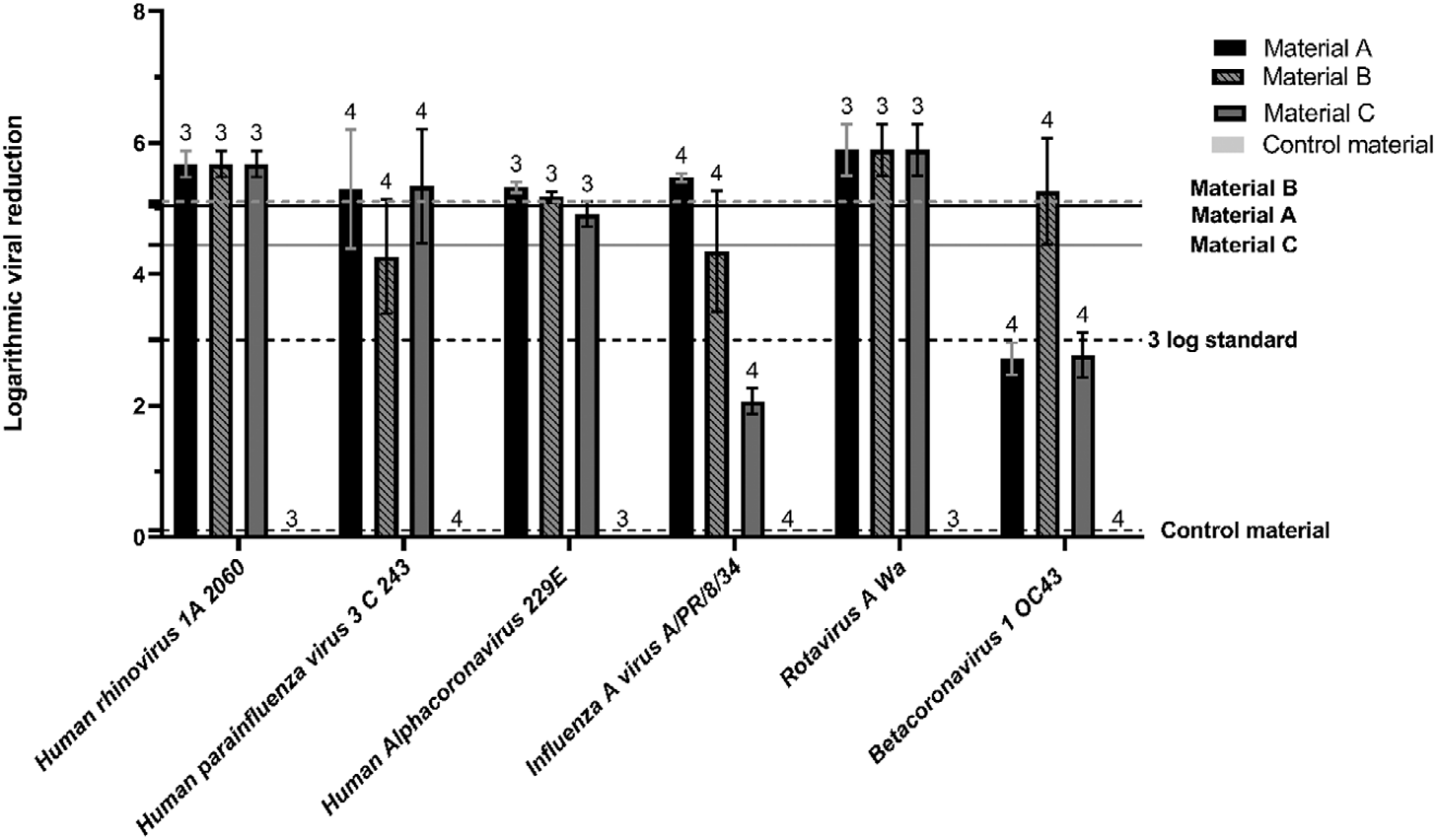
Plaque assay results (*n* = 3) after 1 hour of contact of a viral specimen with the metalized materials and control material are represented as mean ± SD. All viruses present a titer reduction after one hour of exposure to the metalized materials. Three viral species, OC43, HPIV‐3 and PR8 (*n* = 4) present a lower logarithmic reduction as seen for materials A, B, and C. The mean logarithmic virucidal activity of the metalized materials and control was represented as a horizontal line for better visualization and comparison of the data (material A 5.06, material B 5.10 and material C 4.44).

One gastrointestinal virus and five respiratory viruses were tested against the three metalized materials and the unmetalized control. One hour of exposure of a virus to all of the metalized materials resulted in a viral titer reduction (**Figure** **3**), whereas the control material presents no notable viral titer reduction as previously described. Material C however, did not meet ISO standard 18184:2019 (requirement of a reduction greater than 3 log) with titer reductions of 2.06 (PR8) and 2.76 (OC43) (Figure 3). Based on the average logarithmic neutralization, all materials have virucidal activity with material B having a higher efficiency with a viral logarithmic reduction average of 5.10 (SD = 0.54), followed by material A with 5.06 (SD = 0.31) and material C with the lowest logarithmic reduction of 4.44 (SD = 0.36).

### 3.3 Cytotoxicity

#### 3.3.1 The Cytotoxicity of Metalized Materials in HEKa Cells

The BS EN ISO standard 10993–5:2009 method is based on the relative toxic reactivity grade of the cell monolayer via direct or indirect contact with a medical device. To identify if metalized materials confer simultaneous antibacterial/antifungal/antiviral activity and low mammalian cytotoxicity the BS EN ISO standard 10993–5:2009 was followed.

All metalized materials present toxic activity against HEKa cells after two hours of incubation via indirect agar contact. Based on the ISO standard results interpretation guidelines, materials A and B present low to mild toxic activity. The positive control presents severe toxicity on the ISO scale, followed by material C, which presents a higher reactivity grade than the other materials, whereas the control material is non‐toxic (**Table** **1**). Material B has the smallest cytotoxic effect via the indirect method and could be considered safe for handling over a 2 h period as tested.

**Table 1:** Average reactivity gradea) of the cell monolayer (*n* = 3) regarding cytotoxicity of the indirect contact material tested on HEKa cell lines according to ISO10993‐5:2009.

Cell line average	Material A	Material B	Material C	+Ve	Cm
HEKa	1.66	0.33	2.33	4	0

^a)^ The result of a grade greater than 2 is considered to have cytotoxic activity, whereas zero is marked as no reactivity or toxicity to a cell monolayer, one is slight toxicity, two is mild toxicity, three is moderate toxicity and 4 is considered as severe toxic effect.

**Figure 4:**
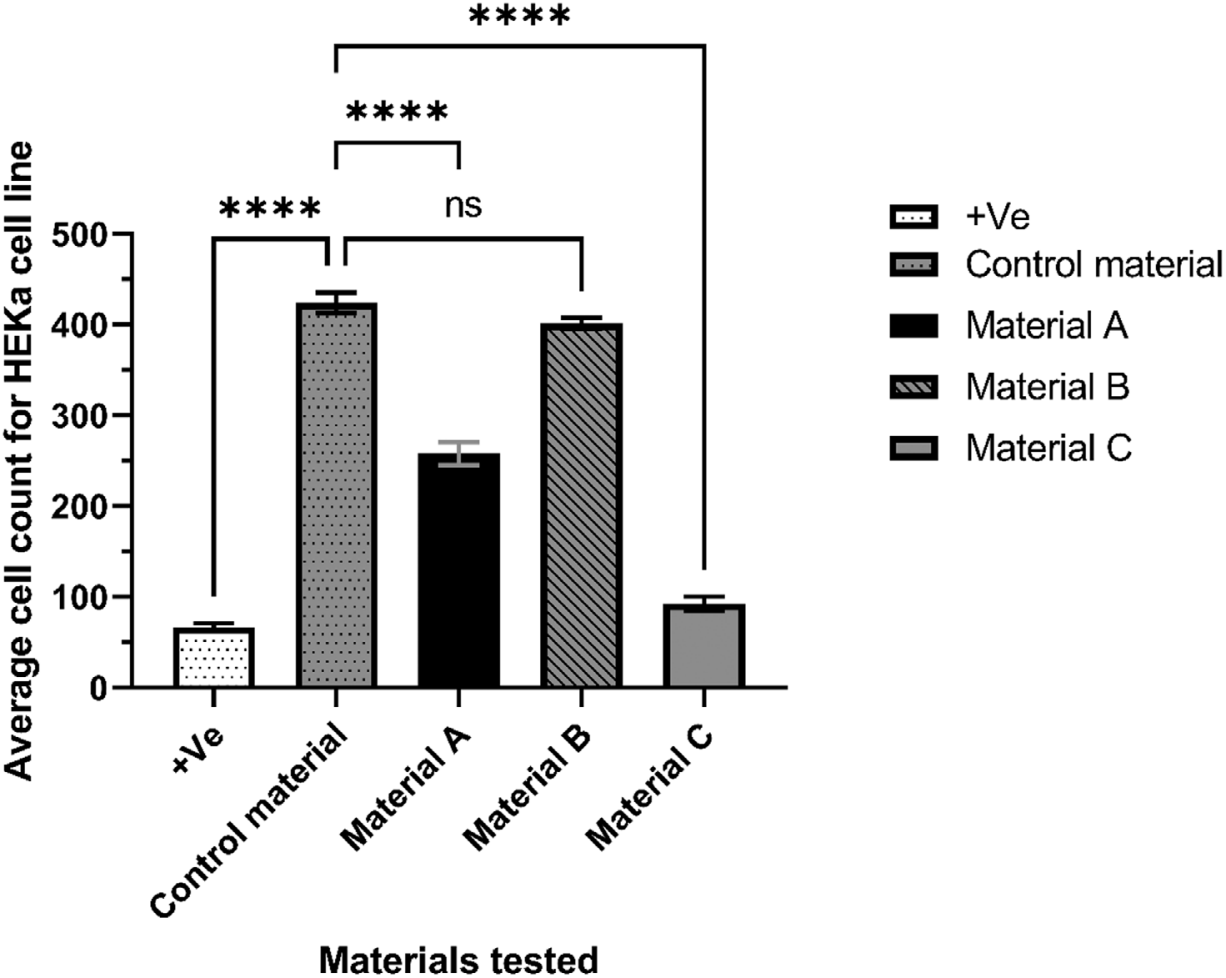
Indirect cytotoxicity test via ISO10993‐5:2009 in HEKa cell line (*n* = 3) showing the average cell count. Statistical significance (*P* < 0.0001) between samples is shown (one‐way ANOVA). The positive control and material C present the lowest cell count followed by material A. Material B, control material and negative control present the highest cell number, resulting in material B being the less cytotoxic of all the metalized materials tested.

The average cell counts (**Figure** **4**) for the indirect cytotoxicity experiments establish the same pattern as observed visually according to the reactivity grade from the ISO standard. The positive control followed by material C presents the smallest cell count presenting a direct cytotoxic effect. The cell count for material A is significantly lower (*P* < 0.0001) compared to the control material, whereas material B shows a cell count virtually identical to that of the control material, with no significant difference (P = 0.20). This series of quantitative results mirrors the results obtained from the qualitative reactivity grade test suggesting that material B has the lowest cell toxicity effect compared with the other 2 metalized materials.

**Figure 5:**
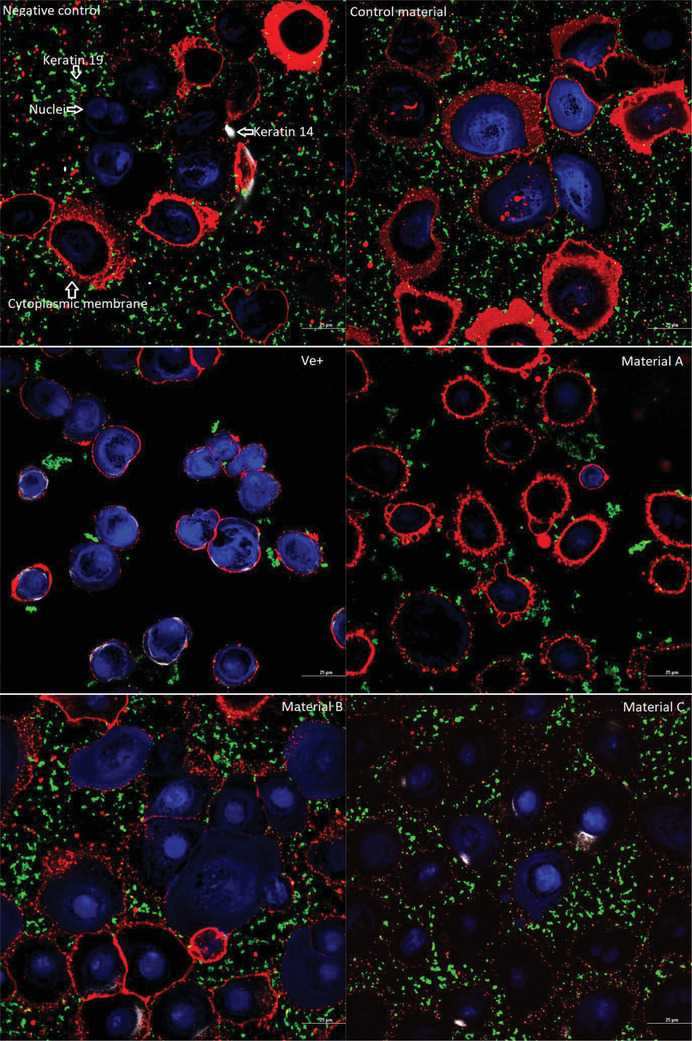
Indirect agar contact cytotoxic testing (*n* = 3) visualized under confocal microscopy images with an oil objective at × 100 magnification of HEKa cell line, fixed with 4% paraformaldehyde. Red/orange stain is based on CellBrite Cytoplasmic Membrane Dye, blue nuclei dye is based on the Live‐or‐Dye Fixable Viability stain, Human Cytokeratin 14 APC‐based antibody stain is white labelled and Human Cytokeratin 19 Alexa Fluor 488‐conjugated antibody is labelled as green. The unmetalized control material and the negative control present no morphological changes, with round and well‐defined borders morphology, nuclei slightly stained due to paraformaldehyde activation and penetration of the cell membrane, with small distribution of keratin 14 and well distribution of keratin 19. The positive control after 2 h of incubation in Distel shows abnormal morphology with bright nuclei and thin cellular membrane, followed by a small amount of keratin 19 and more distribution of keratin 14. Material B presents identical morphology with the control material whereas material A and C show cytotoxic effects.

The confocal images of the HEKa cell line (**Figure** **5**) show the same pattern of results as seen in the indirect agar ISO standard method, where material B had no toxic activity compared to materials A and C. Negative control and unmetalized material present a normal cell morphology, with some stained nuclei, a vast distribution of keratin 19 over the cell monolayer, and a small distribution of keratin 14. The positive control presents direct cell morphology alternation, direct cell rounding and membrane degradation with bright nuclei stain, with no viable cells present, and a lack of keratins. Material C presents a visible distribution of keratin 14 and 19 with a thin cell membrane and pores. Material A shows a slight alternation of cell shape, with an overall rounding and low nuclei intensity, with a noticeable reduction of keratin 19 and 14 in the cell monolayer. Material B presents identical cell distribution, keratin, and morphology with the negative control and unmetalized control material with small pores in the cellular cytoplasmic membrane.

The indirect agar cytotoxicity testing shows a difference in sample behavior, which was confirmed with the confocal microscopy by observing the alterations in cell morphology and cytokeratin distribution between controls and metalized materials. Material B shows lower cytotoxic activity after 2 h of contact, whereas materials A and C present cytotoxic results as observed previously.

#### 3.3.2 Direct Contact Cytotoxicity Testing in 3D Culture

To identify if the same cytotoxic patterns observed in the 2D models are present in a 3D model the HEKa cells were cultured in a 3D scaffold and then exposed to the metalized materials. The 3D cell model showed the same pattern as observed in the other methods of testing (**Figure** **6**), indicating that the control material did not affect the cell morphology or cell proliferation. However, the positive control and material C show a direct alternation of keratinocytes, cell membranes and nuclei. Materials A and B do not present any cytotoxic effect on any cellular structure after the incubation period, compared with control material reinforcing the results obtained from the 2D model (Figure 6). Material C presents similar morphological changes as in the 2D model with low expression of keratin 19 and 14, followed by high signal expression of nuclei and altered cytoplasmic membrane expression. The sectional rendering of the 3D scaffold as a volume (supplementary materials for Z stack recording) shows that material B presents overall less cytotoxic effects over the Z axis, compared to materials A and C.

Figure 6Cytotoxic testing of a 3D model of HEKa cell line analyzed using confocal microscopy with an objective at × 20 magnification using different staining and antibody techniques. Red/orange stain is based on CellBrite Cytoplasmic Membrane Dye (TRITC channel), blue nuclei dye is based on the Live‐or‐Dye Fixable Viability stain (DAPI channel), Human Cytokeratin 14 APC‐based antibody stain is pink labelled (APC channel) and Human Cytokeratin 19 Alexa Fluor 488‐conjugated antibody is labelled as green (FITC channel). The 3D model was recorded via confocal microscopy with an average of 40/50 Z sections of pictures per sample and the maximum intensity projection from Z axes was generated via the software. The unmetalized control material and negative control can be observed without any noticeable cytotoxic effect, whereas the positive control and material C present direct cell damage. Material A and B present no cell damage, normal membrane, and nuclear distribution as the control material.

**Figure:**
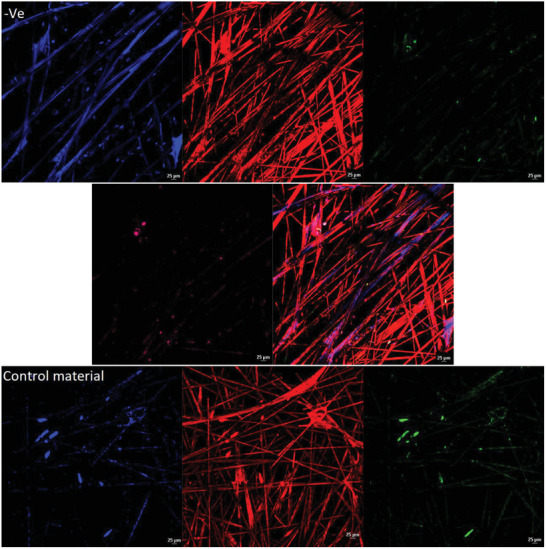

**Figure:**
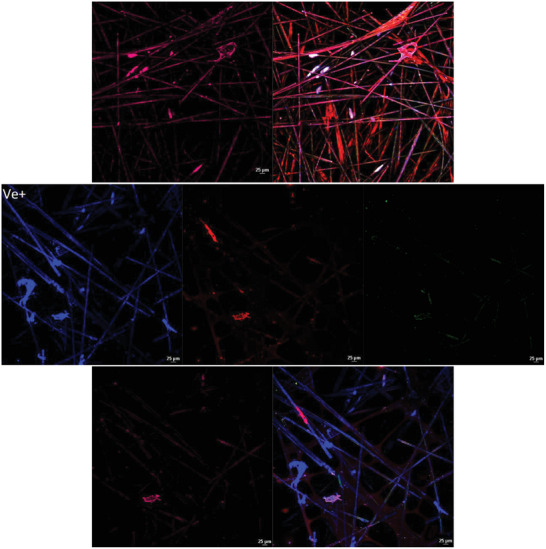

**Figure:**
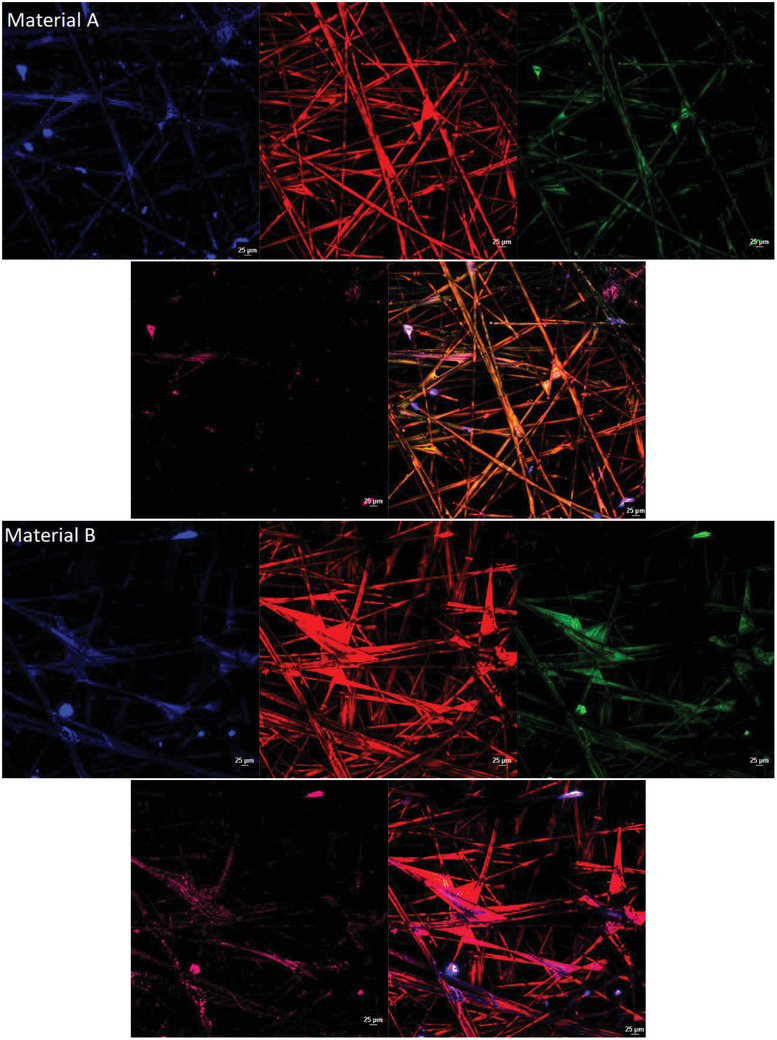

**Figure:**
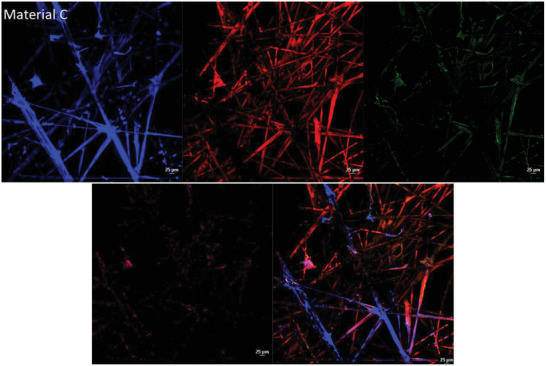

**Figure 7:**
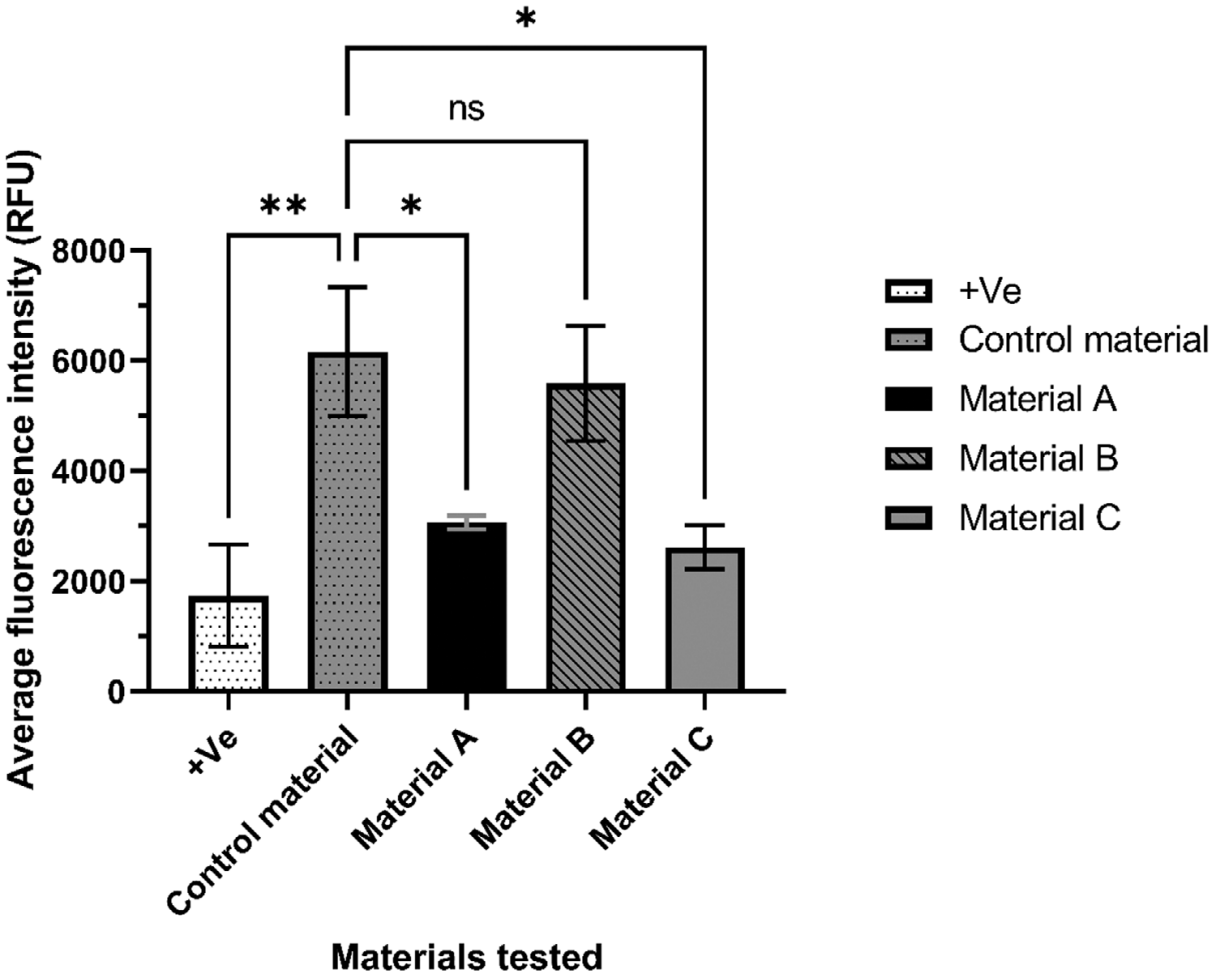
The average intensity of HEKa 2D models was analyzed using a one‐way ANOVA test (*n* = 3). The results indicate that the positive control, as well as materials A and C, exhibit significant differences in cytotoxicity compared to the control material. In contrast, material B does not show a significant difference (*P* = 0.89) in cytotoxicity.

Furthermore, to reinforce the results obtained by calculating the average fluorescence intensity from all results presented via the confocal technique, material B presents similar intensities with the negative control and control material (**Figure** **7**), whereas materials A and C present notable lower values (Figure 7) which can be correlated with the toxic activity of the material. Material B showed no significant difference in average fluorescence intensity compared to the control material, as indicated by a one‐way ANOVA (*P* = 0.89).

### 3.4 Material Aging

**Figure 8:**
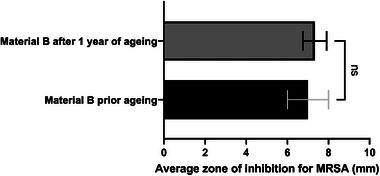
Agar diffusion of unaged and accelerated aged material B against MRSA (*n* = 3), represented as a bar chart. The paired t‐test showed no significant (*P* = 0.74) difference after accelerated aging, resulting in material B having long‐lasting antibacterial efficiency.

Material B conferred broad antimicrobial activity and exhibited the least in vitro cytotoxicity. Therefore, it was selected to undergo accelerated aging to identify if the antimicrobial activity was maintained over time. MRSA isolate was selected as a relevant candidate as it causes substantial healthcare burdens. Material B results were compared with the results obtained after aging (**Figure** **8**), and no significant difference (*P* = 0.74) in the antibacterial activity could be observed, with an inhibition zone average of 7.33 mm against MRSA concluding that the material still presents antibacterial activity after one year of accelerated aging. As observed in previous results, the control material did not present any inhibition zone after aging.

### 3.5 Material Characterization

**Figure 9:**
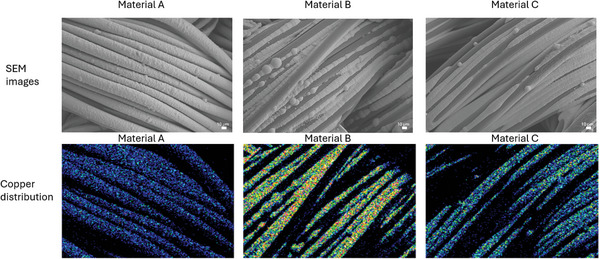
Scanning electron microscopy images of metalized textile and energy dispersion spectroscopy images of copper distribution across the respective areas.

The images of the metalized textile and copper distribution across the textile fibers are presented in **Figure** **9**. These results are consistent with previously reported images of metalized textiles using the same method of preparation.^[^ ^39^, ^40^ ^]^ According to Figure 9, in material A, copper covers the textile threads continuously, while in materials B and C, a significant amount of copper is located between adjacent fibers. This position of the deposited metal film is due to capillary forces that direct catalyst distribution when catalyst ink is printed on the hydrophobic material.^[^ ^48^ ^]^ Similar to previous work,^[^ ^39^ ^]^ no considerable difference was observed in the morphology and copper distribution across materials B and C.

## 4 Discussion

This research describes a comprehensive antimicrobial screen of 3 different metalized polyester textile materials. Material A (immersion), B (ink‐jet printing parallel warp threads) and C (ink‐jet printing parallel weft threads) exhibit strong virucidal and bactericidal effects which could combat pathogenic spread (Figures 2 and 3). However, when considering cytotoxicity data, material B, despite having almost similar pathogen reduction capability exhibits a lower rate of toxicity against primary human cells compared to the other tested materials.

The bactericidal effect of the metalized materials was evident across all 12 isolates tested within 30 min of direct contact (Figure 2). For example, material B from this study achieves bactericidal effects after just 30 min, whereas other studies using copper‐coated textiles have shown antibacterial effects only after prolonged exposure, for instance, *P. aeruginosa* remained viable for up to 5 h of incubation with copper‐coated materials.^[^ ^49^, ^50^, ^51^, ^52^, ^53^ ^]^ Other coating strategies, such as those utilizing high‐power ultrasound,^[^ ^54^ ^]^ have demonstrated antibacterial effects against *E. coli* after a minimum of 7 h of incubation,^[^ ^54^, ^55^ ^]^ the metalized textiles in this study achieve similar effects in a shorter period.

Material B exhibits prolonged antipathogenic activity, maintaining efficacy over an extended shelf life as demonstrated by accelerated aging results (Figure 8) and as seen in other studies.^[^ ^56^, ^57^ ^]^ Further additional in vivo testing is necessary to confirm the pattern of shelf‐life. A key characteristic that sets metalized materials apart from traditional bactericidal chemicals and cleaning methods is their ability to maintain persistent bactericidal effects over time, whereas the effects of chemical treatments may diminish. This enduring bactericidal efficacy of metalized materials may be attributed to the distribution of metabolic activity, blockage of protein synthesis and toxic ion release at elevated concentrations.^[^ ^58^ ^]^ It needs to be considered that, resistance to the surface coatings can be developed. For example, *Candida* species produce biofilms as a protective mechanism against toxic metal concentrations.^[^ ^59^ ^]^ Bacteria can perform multiple survival strategies such as changing the metal ion charges, forming chelates, repulsing ions, bioleaching, and switching to cellular ion efflux pumps.^[^ ^60^ ^]^ Long‐lasting exposure to toxic elements can generate mutations and promote pathogenic resistance, a good example of this is *Staphylococcus aureus* and *E. coli* J53 which established silver resistance after long‐time exposure to the metal.^[^ ^61^, ^62^ ^]^

This is the first known study to demonstrate a broad virucidal effect for all 3 metalized materials in 1 h of contact according to BS EN ISO 18184:2019, with almost complete virucidal activity in most tested viruses (Figure 3). In a crowded environment, the release of infectious particles from a cough (averaging 3 × 10^3^ droplets) or a sneeze (up to 4 × 10^4^ droplets) poses a significant risk of infection.^[^ ^63^ ^]^ The broad‐spectrum virucidal effects demonstrated by the metalized materials in this study suggest promising potential for improving infection control through the application of surface coatings. This approach has the potential to greatly reduce or completely neutralize the risk of infection, depending on the pathogen involved, thereby carrying substantial implications for public health and disease prevention.

Multiple studies use long incubation periods for antiviral effects (more than 3 h) and lack testing against multiple viral specimens.^[^ ^52^, ^64^, ^65^ ^]^ Similar studies which use copper spraying deposition method do not offer a full antiviral pattern in one hour of incubation, for example, Human Alphacoronavirus 229E and Human Rhinovirus require 2 h of incubation for virucidal effects.^[^ ^66^ ^]^

Research on metalized fabrics using advanced equipment such as a DC magnetron sputtering system has shown that copper‐modified textiles achieve a 2‐log antiviral effect against the Influenza virus following a 2 h incubation period.^[^ ^67^ ^]^ In contrast, materials A and B investigated in this study display over 4‐log viral reduction within just one hour. Similar results as observed in this study were present for Human Alphacoronavirus 229E, where the textile generation was accomplished at an industrial scale showcasing that large‐scale manufacturing for virucidal materials is possible.^[^ ^68^ ^]^

During the Sars‐CoV‐2 pandemic, multiple papers released antiviral claims without antiviral testing.^[^ ^69^, ^70^ ^]^ To remove any uncertainty, in this study a large pallet of species were tested following the BS EN ISO 18184:2019 and a broad virucidal spectrum effect was ensured in just 1 h of contact as seen for material B (Figure 3) with an average of 5.10 logarithmic viral reduction. However, it needs to be noted that comparing bactericidal/virucidal results from different studies can be complex and challenging due to variations in the coating generation process and materials, which impacts the coating properties and therefore activities.^[^ ^52^, ^53^, ^54^, ^55^, ^71^, ^72^ ^]^ This study exclusively tested RNA‐based viruses and it should be considered that DNA viruses such as adenoviruses,^[^ ^73^ ^]^ or canine parvovirus,^[^ ^74^ ^]^ should be included in future testing, as DNA viruses possess distinct structures and envelopes, and they tend to be more resilient in the environment.^[^ ^75^ ^]^ As further work, multiple other species should be tested in different scenarios such as in vivo action in clinical settings or crowded environments.

Keratins 14 and 19 are primary indicators for the structural integrity of cells and provide a model for correct cellular configuration in vitro. It should be noted that keratin expression can be modified by cytotoxic reactions and may rely on primary or secondary metabolic or signaling processes as part of the cellular defense mechanism.^[^ ^76^, ^77^ ^]^ Keratin 19 helps maintain the structural integrity of epithelia and envelopes the developing epidermis.^[^ ^78^ ^]^ Keratin 14 assembles into a strong filament network to help attach the epidermis to the underlying layer of skin, maintain shape and provide resistance against external factors.^[^ ^79^ ^]^ Overall, from the results obtained (Table 1), 2 out of 3 materials have toxic activity which either destabilizes or alters the conformational change of the membrane layer (Figure 5) or directly interacts with the expression and distribution of keratin 19 and 14 (Figure 6). In 2D models, material B shows undetectable changes in the keratin layers and no cell morphology alternation (Figure 5) compared with materials A and C which have clear toxic activity against the cell monolayer. The 3D model of materials A and B (Figure 6) shows no cell damage on the scaffold layers, resulting in no direct contact damage or diffusion from the material tested. Furthermore, from the average fluorescence intensity results (Figure 7) and the 3D rendering, a difference in cell cytotoxicity could be observed between the metalized materials tested. Material B biocompatibility with human skin enables promising applications in daily circumstances, as 2 h contact with human skin would not have direct toxic activity.

The metalized textile materials were made using two kinds of catalysts: Pd (material A) and Cu/Ag (materials B and C). A difference in antipathogenic effect and cytotoxicity was expected between these two groups, as it is known that the nature of the catalyst significantly affects the quality and properties of electroless‐plated copper.^[^ ^40^, ^47^ ^]^ However, materials B and C were prepared using the same catalyst, which was deposited along warp and weft threads, respectively. A previous study shows that the way of catalyst deposition does not affect film morphology or mass gain,^[^ ^39^ ^]^ meaning both materials have a similar amount of copper. However, it affects the film's mechanical and electrical properties, which was previously attributed to the difference in catalyst absorption between materials due to the difference in thread count (there are twice as many warp threads as weft). Nevertheless, this does not explain why the antipathogenic/cytotoxic properties of materials B and C are so different. A study showed that the antimicrobial properties of the copper layer can be affected by grain size, lattice defects, and the presence of specific reactive oxygen species.^[^ ^80^ ^]^ Further research needs to be accomplished to understand the structural and chemical differences between the two materials and how this interaction impacts overall activity.

To address future population and ecosystem changes, it is crucial to implement strategies that prevent pathogen transmission along transport and trade routes;^[^ ^16^ ^]^ however, the material developed in this study requires further in vivo cytotoxicity testing due to possible material leaching or long‐time environmental degradation. Developing a long‐lasting antipathogenic and biocompatible material is key to challenge and reduce pathogen transmission, HAI and AMR.^[^ ^53^, ^81^, ^82^, ^83^ ^]^ Considering the novel data presented, this research highlights the effective virucidal and bactericidal properties of metalized textiles, demonstrating rapid action and broad application, with potential use in clinical and high‐demand environments like shops, markets, and airports. These materials offer versatile solutions to reduce transmission and infection rates, potentially curbing hospital‐acquired infections and associated costs. Material B shows biocompatibility and possible versatile application in healthcare settings, such as in textiles for curtains, bedding, and personal protective equipment, and in medical implements like X‐ray vests, surgery trays, medical crepe paper or cleaning objects such as mops. Additionally, their textile‐based nature allows for tailored solutions in personalized medicine, with wider‐scope applications in bioelectronics (not further tested in this research);^[^ ^39^, ^40^ ^]^ for example, the material could be integrated as an antiseptic for a rapid heart rate monitoring system. Looking forward, these materials are crucial in addressing current and novel pathogenic threats, including pandemics, amidst ongoing urbanization and environmental changes.^[^ ^16^, ^84^ ^]^

## 5 Conclusion

To conclude, for the first time, all three materials tested have bactericidal and virucidal activity against a diverse array of clinically relevant bacterial and viral species, in a short time, whereas material B (ink‐jet printing catalyst parallel warp threads) exhibits a low cytotoxic effect in the 2D and 3D models resulting in biocompatibility with primary human keratinocytes. The multipurpose textile material B could be used in personalized medicine and considering the unprecedented results obtained, material B could have great potential in pathogen prevention and control. In clinical facilities material B could lower nosocomial infections whereas in daily circumstances the material could be used in infection hot spots directly lowering the infection rate and transmission patterns.

## Conflict of Interest

The authors declare no conflict of interest.

## Supporting information



Supporting Information

## Data Availability

The data that support the findings of this study are available from the corresponding author upon reasonable request.
