# Supplementary material for: Virucidal and Bactericidal Properties of Biocompatible Copper Textiles
Source: Glob Chall. 2025 Jan 27;9(3):2400346. doi: 10.1002/gch2.202400346 (PMC11891573; doi:10.1002/gch2.202400346)
Supplement: Supplementary file 1 — Supporting Information [file GCH2-9-2400346-s001.docx]

**Table S1. Microbiological isolates, culture number, growth media and conditions.**

| **Name/Species** | **Culture number** | **Conditions for growth on solid media:** |
| --- | --- | --- |
| *Candida albicans* | ATCC 76615 | Potato dextrose agar (PDA) 24°C to 26C |
| *Enterobacter cloacae* | Lab strain | Nutrient agar, 37°C, aerobic |
| *Enterococcus faecalis* | ATCC 51299 | nutrient/blood agar,37, facultative anaerobe |
| *Escherichia coli* B | ATCC 23848 | Nutrient agar 37°C, aerobic |
| *Escherichia coli* K12 | NCTC 12923 | Nutrient agar 37°C, aerobic |
| *Listeria monocytogenes* | ATCC 7646 | Brain Heart Infusion Agar/Broth 37°C |
| *Proteus mirabilis* | NCTC BS711 | Brain Heart Infusion Agar/Broth 37°C |
| *Pseudomonas aeruginosa* | ATCC 27853 | Trypticase Soy Agar/Broth,37°C |
| *Salmonella enteritidis* | ATCC 13076 | Nutrient agar or nutrient broth 37°C |
| *Salmonella typhimurium* | Lab strain | Nutrient agar or nutrient broth 37°C |
| *Serratia marcescens* | Lab strain | Nutrient agar or nutrient broth 37°C |
| *Staphylococcus aureus* MRSA | ATCC 43300 | Trypticase Soy Agar/Broth,37°C |

**Table S2. Cell line catalogue number, origin, growth media, CO_2_, and temperature.**

| **Specimen** | **Cell type and origins** | **Growth Media, CO_2_, and temperature** |
| --- | --- | --- |
| A549 (ATCC, cat: CRM-CCL-185) | Human epithelial lung cells- 58 years old male | EMEM/DMEM 10%FBS– 5% CO_2_, 37°C |
| BS-C-1 (ATCC, cat: CCL-26) | African green monkey kidney epithelial | DMEM (Corning, cat:10-013-CM) 10%FBS– 5% CO_2_, 37°C |
| HeLa (ATCC, cat: CCL-2) | Human epithelial cervix cells- 31 years old female | EMEM/DMEM 10%FBS– 5% CO_2_, 37°C |
| MA-104 clone 1 (ATCC, cat: CRL-2378.1) | African green monkey kidney epithelial cloned cells | EMEM 10%FBS– 5% CO_2_,37°C |
| MDCK (ATCC, cat: CCL-34) | Canine kidney epithelial cells- adult, female cocker spaniel | DMEM 10%FBS– 5% CO_2,_ 37°C |
| MRC-5 (ATCC, cat: CCL-17) | Human lung fibroblast- male 14 weeks embryo | EMEM (Corning, cat:10-009-CV) 10%FBS – 5% CO_2,_ 37°C |
| Primary adult epidermal keratinocytes (HEKa) (ATCC, cat: PCS-200-011) | Primary adult epidermal keratinocytes | Dermal cell basal medium (ATCC, cat: PCS-200-030), keratinocyte growth kit (ATCC, cat: PCS-200-040),5% CO_2,_37°C |

**Table S3. Viral conditions for each specimen, including catalogue number, cell host, temperature, media, period of infection and supplements necessary for viral propagation.**

| **Name/Species** | **Culture number** | **Growth conditions and host** |
| --- | --- | --- |
| *Beta coronavirus 1 OC43* | ATCC, cat: VR-1558 | *MRC-5/A549*, EMEM, 0% FBS, 33°C, 3-5 days |
| Human *Alpha coronavirus* 229E | ATCC, cat: VR-740 | *MRC-5/A549*, EMEM, 0% FBS, 35°C, 3-5 days |
| Human parainfluenza virus 3 *C 243*, HPIV-3 | ATCC, cat: VR-93 | *HeLa*, EMEM/DMEM,2% FBS ,33°C, 2-4 days |
| Human rhinovirus 1A *2060* | ATCC, cat: VR-1559 | *HeLa*, EMEM/DMEM,2% FBS ,33°C, 1-3 days |
| Influenza A virus (H1N1) A/PR/8/34 cell culture adapted PR8 | ATCC, cat: VR-1469 | *MDCK*, DMEM,2% FBS ,37°C, 1-3 days, TPCK 1 μg/ml during culturing |
| Rotavirus *A Wa* | ATCC, cat: VR-2018 | *BS-C-1/MA-104*, EMEM/DMEM,2% FBS ,37°C, 1-2 days, 1-hour activation 37°C with porcine pancreatic trypsin 10 μg/ml (Sigma-Aldrich, cat: T4799), during culturing 2 μg/ml |
